# *Cistanche tubulosa* (Schenk) Wight Extract Enhances Hindlimb Performance and Attenuates Myosin Heavy Chain IId/IIx Expression in Cast-Immobilized Mice

**DOI:** 10.1155/2019/9283171

**Published:** 2019-10-22

**Authors:** Yoshiyuki Kimbara, Yutaka Shimada, Tomoharu Kuboyama, Chihiro Tohda

**Affiliations:** ^1^Division of Neuromedical Science, Institute of Natural Medicine, University of Toyama, 2630 Sugitani, Toyama 930-0194, Japan; ^2^Department of Japanese Oriental Medicine, Graduate School of Medicine and Pharmaceutical Sciences, University of Toyama, 2630 Sugitani, Toyama 930-0194, Japan

## Abstract

Skeletal muscle atrophy is encountered in many clinical conditions, but a pharmacological treatment has not yet been established. *Cistanche tubulosa* (Schenk) Wight is an herbal medicine used in traditional Japanese and Chinese medicine. In the current study, we investigated the effect of *C. tubulosa* extract (CTE) on atrophied muscle *in vivo*. We also investigated hindlimb cast immobilization in mice and devised a novel type of hindlimb-immobilizing cast, consisting of sponge-like tape and a thin plastic tube. Using this method, 3 out of 4 groups of mice (*n* = 11 for each group) were cast-immobilized in the hindlimbs and administered CTE or vehicle for 13 days. A sham procedure was performed in the mice of the fourth group to which the vehicle was administered. Next, the triceps surae muscles (TS) were excised. To analyze the effect of the novel cast system and CTE administration on muscle atrophy, we evaluated TS wet weight and myofiber cross-sectional area (CSA). We also determined MyHC IId/IIx expression levels by western blotting, since their increase is a hallmark of disuse muscle atrophy, suggesting slow-to-fast myofiber type shift. Moreover, we performed two tests of hindlimb performance. The novel cast immobilization method significantly reduced TS wet weight and myofiber CSA. This was accompanied by deterioration of hindlimb function and an increase in MyHC IId/IIx expression. CTE administration did not alter TS wet weight or myofiber CSA; however, it showed a trend of amelioration of the loss of hindlimb function and of suppression of the increased MyHC IId/IIx expression in cast-immobilized mice. Our novel hindlimb cast immobilization method effectively induced muscle atrophy. CTE did not affect muscle mass, but suppressed the shift from slow to fast myofiber type in cast-immobilized mice, ameliorating hindlimb function deterioration.

## 1. Introduction

The pathophysiological conditions associated with skeletal muscle atrophy include sarcopenia [1], cachexia [2], and glucocorticoid-induced skeletal muscle atrophy [3]. Skeletal muscle atrophy is also caused by prolonged disuse [4], which can be due to bed rest [5], application of casts for the treatment of fractures or ligament injuries [6], or spaceflight [7]. Since various diseases can cause this condition, it affects a large percentage of the population.

Adequate exercise and nutritional therapies are essential to treat muscle atrophy [8]. In addition, some studies have reported that pharmacological countermeasures, including selective androgen receptor modulators, human monoclonal antibodies against myostatin, and selective ghrelin receptor agonists, are effective [9]; however, these approaches are not yet established.

The muscle atrophic conditions described above are accompanied by a shift in the myofiber type profile and a reduction of muscle mass. Muscle disuse caused by loss of neural influence and mechanical unloading induces a slow-to-fast myofiber type shift, and a change in the myosin heavy chain (MyHC) isoform profile [10], signaled by the increment of MyHC IId/IIx expression levels [11, 12]. A fast-to-slow myofiber type shift is instead induced by glucocorticoid administration, cachexia, sepsis, and other factors [10].

Cistanche is an herbal medicine originating from *Cistanche deserticola* Y. C. Ma, *C. tubulosa* (Schenk) Wight, or *C. salsa* (C. A. Mey) Beck, according to the Japanese pharmacopoeia [13]. In traditional Japanese (Kampo) and Chinese medicine, it is used to treat “kidney-deficiency syndrome,” characterized by infertility, systemic muscle weakness (especially in the back and lower limbs), and bone weakness [14, 15]. It is known to contain various constituents, including acteoside, echinacoside, and isoacteoside [16]. The extract of this herb, or its chemical constituents, has shown cardio-, hepato-, and neuroprotective effects; antiaging, antifatigue, vasodilatory, and aphrodisiac effects; antioxidant and anti-inflammatory activity; and the ability to improve memory and learning [15, 16]. Nevertheless, the efficacy of this herb to prevent or treat muscle wasting diseases has not been confirmed. Therefore, in the present study, we investigated the effects of *C. tubulosa* extract (CTE) as a treatment for muscle atrophy.

To this end, we used hindlimb cast immobilization to induce muscle atrophy in mice, focusing, in particular, on the materials used in the cast. A variety of cast systems have been reported, including plaster of Paris® casts [17], fiberglass casts [18], polypropylene microcentrifuge tubes [19], restraint suit-like devices made of cotton and steel mesh [20], plastic pipettes with proximal sponge padding [21], casts made of nonelastic bandage tape and vinyl-coated steel wire [22], wire insertion combined with metal plates [23], and the use of a surgical skin stapler [24]. An ideal cast should be minimally invasive and nontraumatic to avoid inflammatory changes because inflammation can induce muscle atrophy [25]. In addition, hindlimb performance tests with the cast should be possible at the final stages of the experiment. Furthermore, the cast should be easy to handle without special knowledge or skills; it should be light, inexpensive, easy to purchase, and should not require special devices for application and removal.

Different animal models of muscle wasting have been reported. Hindlimb unloading has been used frequently [11, 12] but has several problems, including tail necrosis, which can induce systemic inflammation [25]; deviation of body fluids to the caudal side, resulting in abnormalities in hindlimb blood vessels [26]; extraordinary microgravity on the hindlimb, which can strongly affect protein expression, including that of mitochondrial enzymes [27]. All these factors can influence the course of muscle wasting. A different method induces muscle atrophy by depriving the muscle of neuronal inputs (for example, *via* sciatic nerve transection) [28]; since muscles are subject to trophic factors from terminal neurons [29], this method, which leads to an unusual near-total denervated status, can alter the process of muscle atrophy. Animals that are subject to systemic perturbations that evoke muscle atrophy, such as diabetes mellitus [30], renal failure [31], corticosteroid administration [32], and aging or accelerated senescence [33, 34], can be used as models of human diseases; however, these systemic conditions can also interfere with the process of muscle atrophy.

Thus, we selected hindlimb cast immobilization to induce muscle atrophy, and we sought to create a novel method to perform it in mice, by which we investigated the effects of CTE on muscle atrophy.

## 2. Materials and Methods

### 2.1. Plant Material and Extraction

CTE was acquired from Alps Pharmaceutical Industry Co., Ltd. (Hida, Gifu, Japan). *C. tubulosa* fleshy stems were collected from Shinjang Uyghur Aptonom Rayoni, People's Republic of China. A total of 193 kg of mixed powders of *C. tubulosa* was immersed in 1,883 l of 30% ethanol. The mixture was refluxed for 2 h at 55–64°C. The extract yield was 7.2% and contained 3.36% acteoside and 12.72% echinacoside.

### 2.2. Mice

Adult ddY (12 weeks old) male mice (Japan SLC, Shizuoka, Japan) were housed separately in plastic cages and maintained at a 12-h light/dark cycle in a temperature-regulated (25 ± 2°C) vivarium. All animals were allowed *ad libitum* access to water and chow (Labo MR stock; Nosan Corporation, Yokohama, Kanagawa, Japan).

All animal experiments and protocols were carried out in accordance with the Guidelines for the Care and Use of Laboratory Animals of the Sugitani Campus of the University of Toyama. The approval number for the animal experiments is A2016INM-3. All efforts were made to minimize the number of animals used.

### 2.3. Induction of Hindlimb Muscle Atrophy and CTE Administration

**Figure 1:**
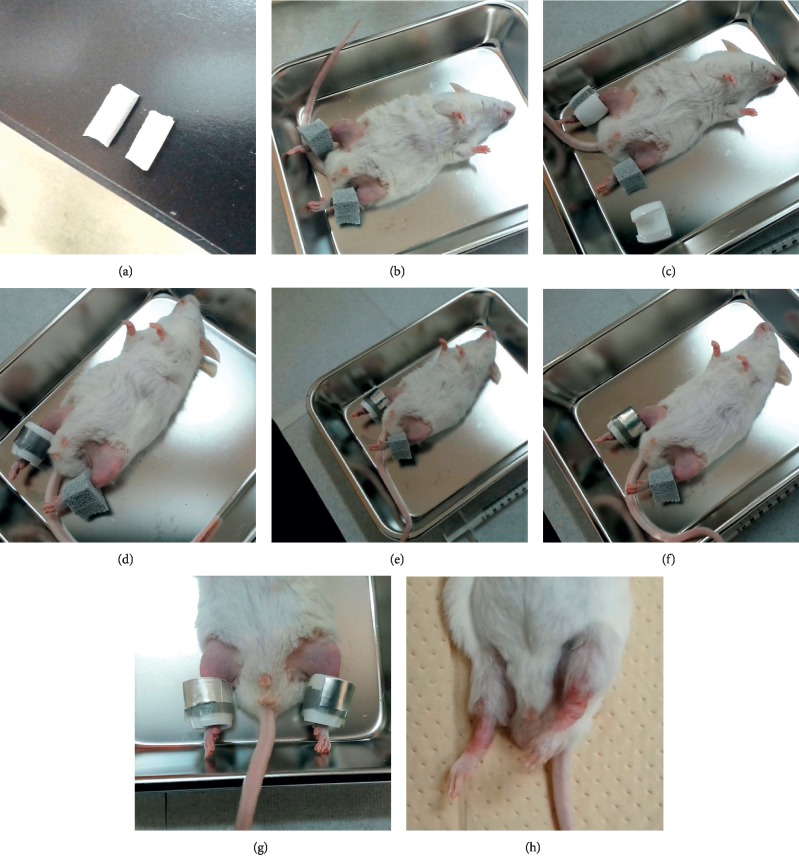
Stepwise hindlimb immobilization process (a)–(g): (a) step 1: cut a 25 mm-long strip of a EPDM rubber foam sealing tape; (b) step 2: hold a hindlimb in the ankle plantar position and wrap it at the ankle joint with the strip; (c) step 3: cut a soft PVC insulation cap and cover the sealing tape cuff with the double-sided tape; (d) step 4: gather the cut ends of the cap and cut the waterproof high-performance adhesive tape to approx. 48 mm in length and fix the gathered ends; (e) step 5: put a small piece (approx. 30 mm long) of an aluminum foil tape on the front side of the upper end of the cap; (f) step 6: cut the vinyl electric insulation tape to approx. 40 mm in length and wrap the cap; (g) step 7: perform the same procedure on the contralateral hindlimb; and (h) general appearance of the hindlimbs at the end of the experiment, after cast removal.

Mice were divided into 4 groups (*n* = 11 per group): at day 2, all mice were anesthetized, and their hair was removed from the thigh to the ankle with a depilatory cream (Epilat cream; Kracie, Tokyo, Japan). Next, 3 out of 4 groups cast (+) groups were cast-immobilized according to the following procedure, as shown in Figures 1(a)–1(g):

 
*Step 1*. Cut a EPDM rubber foam sealing tape (Cemedine Corporation, Tokyo, Japan) into a 12 × 8 × 25 mm strip (Figure 1(a)).
 
*Step 2*. Hold a hindlimb in the ankle plantar position and wrap it very gently at the ankle joint with a sealing tape (Figure 1(b)).
 
*Step 3*. Cut a soft PVC insulation cap TIC-22 (Nichifu Corporation, Osaka, Japan), and cover the sealing tape cuff. Use double-sided tape (PRO SELF handy cut double-sided tape J-1300; Nitoms Inc., Tokyo, Japan) to make them adhere (Figure 1(c)).
 
*Step 4*. Gather the cut ends of the cap; cut waterproof high-performance adhesive tape (power tape; Asahipen Corporation, Osaka, Japan) to approx. 4 × 48 mm size and fix the gathered ends (Figure 1(d)).
 
*Step 5*. Put a small piece (approx. 5 × 30 mm) of an aluminum foil tape (AL-30; Nichiban Corporation, Tokyo, Japan) on the front side of the upper end of the cap (Figure 1(e)).
 
*Step 6*. Cut the plastic tape (vinyl electric insulation tape no. 21; Nitto Denko Corporation, Osaka, Japan) to approx. 19 × 40 mm, and wrap the cap (Figure 1(f)).
 
*Step 7*. Perform the same procedure on the contralateral hindlimb (Figure 1(g)).

The immobilization period used in the present study was 13 days, and casts were replaced if one or more of the following conditions occurred: severe pedal edema, hindlimb necrosis, severe skin ulcerations, loosening of the cast due to destruction, or cast dislocation from the ankle. On the remaining group, only depilation was performed (sham procedure), and vehicle (physiological saline) was administered for 13 days from the day following depilation (day 2). Amongst the 3 cast (+) groups, 2 were administered CTE (50 and 100 mg/kg/day; cast (+)/CTE-50 group and cast (+)/CTE-100 group), and one was administered vehicle (cast (+)/vehicle group), also from day 2. CTE was dissolved into physiological saline and administered daily *via* oral gavage for 13 days.

### 2.4. Evaluation of Hindlimb Function and Hindlimb Muscle Strength

At day 14, the mice of the cast (+) group were anesthetized again after CTE administration, and the casts were removed. The next day (day 15), all mice were tested for hindlimb function and muscle strength, using the following two tests:

#### 2.4.1. Measurement of Gait Speed

A 15 mm wide square wood platform was held horizontally approximately 50 cm from the ground. The mouse was then placed on the platform, and gait speed was assessed as the animal traversed the platform by manually recording using stopwatch the time to walk a distance of 50 cm. Beforehand, each mouse had been habituated to the platform by traversing it twice as a training session.

#### 2.4.2. Foot-Fault Test

To evaluate hindlimb muscle strength, we performed the “foot-fault test,” in which each mouse was placed on the platform again, and the number of hindlimb slips from the platform was tabulated. This test is a modified version of the static rod test, in which aerial rods are fixed using a G-clamp and mice are made to traverse them [35]. Foot-fault testing was performed three times, and the mean number of footfalls across the three sessions was recorded. There was a rest interval of at least 15 minutes between sessions.

### 2.5. Histological and Morphometric Analyses of the Triceps Surae Muscle

Following the evaluation of hindlimb function, mice were deeply anesthetized and euthanatized by physiological saline perfusion. Next, the triceps surae (TS) muscles were excised from both sides of the hindlimbs; samples from the left hindlimb were fixed in 4% paraformaldehyde in PBS solution for 24 h, then transferred into 30% sucrose solution in PBS, and incubated for cryoprotection and cryosectioning. Following this, 12-*μ*m thick sections were cut along the minor axis from the midbelly region of each muscle using a cryostat (Leica 3050S; Leica systems, Nußloch, Germany). Seven to ten sequential sections were mounted on antistrip-coated glass slides (MAS-GP Type A, Matsunami Glass Industry, Osaka, Japan), immediately dried using a hairdryer, stored at −30°C, and stained with hematoxylin-eosin staining (H-E staining, ScyTek Laboratories, Logan, Utah, United States). One representative section per animal, in which the short-axis slice of each myofiber was clearly visible, was selected, and its cross-sectional area (CSA) was measured using Image J (National Institutes of Health, Bethesda, Maryland, United States).

### 2.6. Western Blotting

Total protein from the right TS was extracted by the following method. The right TS was minced using scissors and immediately snap-frozen in liquid nitrogen, stored at −30°C, and later homogenized to make a tissue lysate. Thawed muscles were incubated ice-cold for 30 min in 2 mL M-PER mammalian protein extract solution (Pierce Biotechnology, Rockford, Illinois, USA) and then homogenized. Homogenates were then centrifuged at 4°C and 4000 g for 10 min, and supernatants were collected.

10 *μ*g of total protein in each muscle lysate was loaded and separated by sodium dodecyl sulfate-polyacrylamide gel electrophoresis (SDS-PAGE), followed by electrotransfer onto a nitrocellulose membrane (0.2 *μ*m, #1620112, Bio-Rad Laboratories, Hercules, California, United States). Nonspecific binding was blocked with 5% nonfat dry milk (skim milk powder, 190-12865, FUJIFILM Wako Pure Chemical Industries, Osaka, Japan) in Tris-buffered saline with 0.05% Tween 20® (TBS-T). The membranes were then incubated overnight at 4°C with a primary antibody dissolved in Can Get Signal immunoreaction enhancer solution 1 (Toyobo, Osaka, Japan). After agitating and rinsing with TBS-T, the membranes were incubated for 1 h at room temperature with a horseradish peroxidase- (HRP-) conjugated secondary antibody diluted in Can Get Signal immunoreaction enhancer solution 2 (Toyobo, Osaka, Japan). Signals on the membranes were detected via electrochemiluminescence using luminol. Densitometric analysis was then performed using the CS Analyzer software (Atto, Tokyo, Japan). The antibodies were chosen and diluted as follows: mouse anti-MyHC IId/IIx monoclonal antibody (1 : 1000, clone A4.1025, #05-716, Merck Millipore, Burlington, Massachusetts, United States), mouse anti-GAPDH loading control monoclonal antibody (1 : 3500, G041 abm, Vancouver, Canada), and HRP-conjugated goat antimouse IgG polyclonal secondary antibody H&L (1 : 2000, Abcam, Cambridge, United Kingdom).

### 2.7. Statistical Analyses

All results are expressed as mean ± standard error of the mean (SEM). The statistical significance of the comparisons among groups was verified by one-way ANOVA and post hoc Dunnett's tests or Tukey's HSD test; the Kruskal–Wallis test and post hoc Dunn's tests; or two-way ANOVA with repeated measures and post hoc Dunnett's test. Smirnov–Grubb's test was used to exclude outliers. For all statistical evaluations, *p* values less than 0.05 were considered statistically significant, and all analyses were performed using GraphPad Prism 6 (GraphPad Software, La Jolla, California, United States).

## 3. Results

### 3.1. Animal Health and Effect of CTE on Body Weight

**Figure 2:**
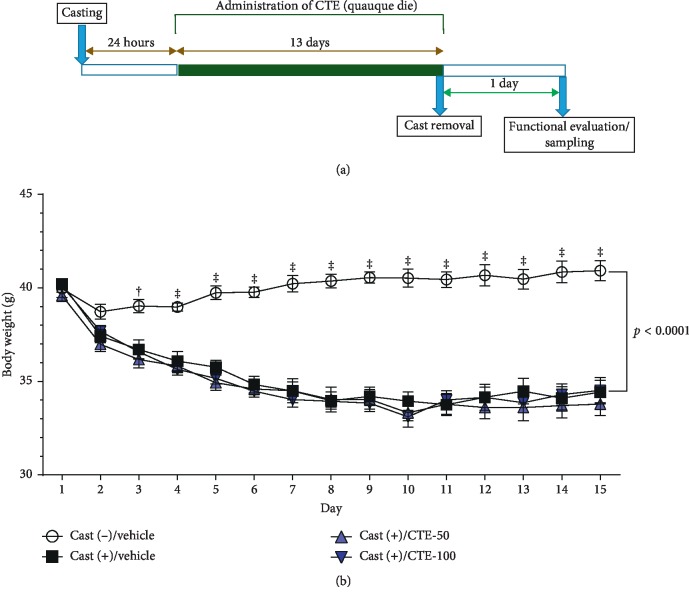
Experimental schedule and body weight trajectory. (a) Time course of the experimental schedule. (b) Average daily body weight of each group over the experiment period. Data are shown as mean ± SEM (*n* = 9–11). Statistical analysis was performed by two-way ANOVA with repeated measures and post hoc Dunnett's test. Interaction: *F* (42, 518) = 13.41. ^†^*p* < 0.01 and ^‡^*p* < 0.0001 vs. cast (+)/vehicle group.

In visual appearance, the hindlimbs of cast-immobilized mice were all atrophic, as compared with those of the cast (−)/vehicle group mice. Cast immobilization resulted in significant losses of body weight by the end of the study phase. By contrast, body weight changes were not significant in cast (−)/vehicle group mice. The two cast (+)/CTE groups exhibited no significant changes in body weight when compared with the cast (+)/vehicle group (Figure 2).

Severe complications in the hindlimbs of immobilized mice, such as skin necrosis or gangrene, were not observed nor was abnormal behavior in the vivarium. However, mild complications, including foot edema, superficial skin ulcerations, and rubor of the hindlimbs, were seen in all cast-immobilized mice (Figure 1(h)). Loosening and dislocation of the cast, leading to cast replacement, were also observed in most cases.

### 3.2. Effect of CTE on Muscle Strength and Muscle Performance

**Figure 3:**
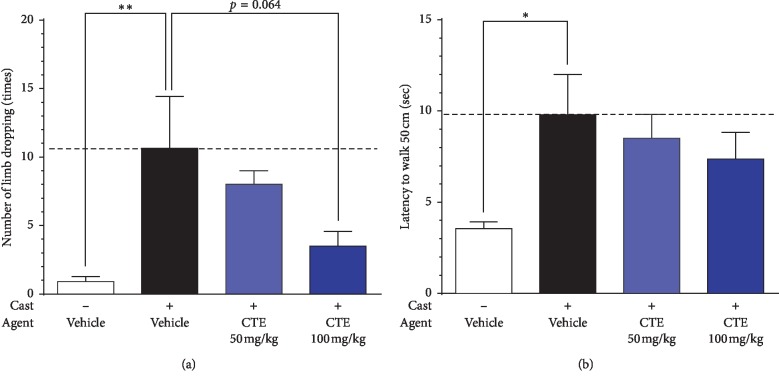
Indices of muscle performance and muscle strength. (a) Number of limb slips from a square platform in the foot-fault test. (b) Gait speed. Data are shown as mean ± SEM (*n* = 9–11). Statistical analysis was performed by one-way ANOVA and post hoc Dunnett's test. ^*∗*^*p* < 0.05 and ^*∗∗*^*p* < 0.01 vs. cast (+)/vehicle group.

The cast (+)/vehicle group exhibited significantly increased numbers of hindlimb slips in the foot-fault test (Figure 3(a)), and gait speed was significantly slower than that in the cast (−)/vehicle group (Figure 3(b)).

CTE treatment resulted in a dose-dependent tendency towards decreased hindlimb slips. In the cast (+)/CTE-100 group, treatment tended to reduce (*p*=0.0604) hindlimb slips when compared with the cast (+)/vehicle group (Figure 3(a)). As for gait speed, both cast (+)/CTE-50 and cast (+)/CTE-100 groups exhibited no significant differences in gait speed compared with the cast (+)/vehicle group, though there was a slight, nonsignificant dose-dependent trend towards faster speed (Figure 3(b)).

### 3.3. Wet Weight of TS and Myofiber CSA

**Figure 4:**
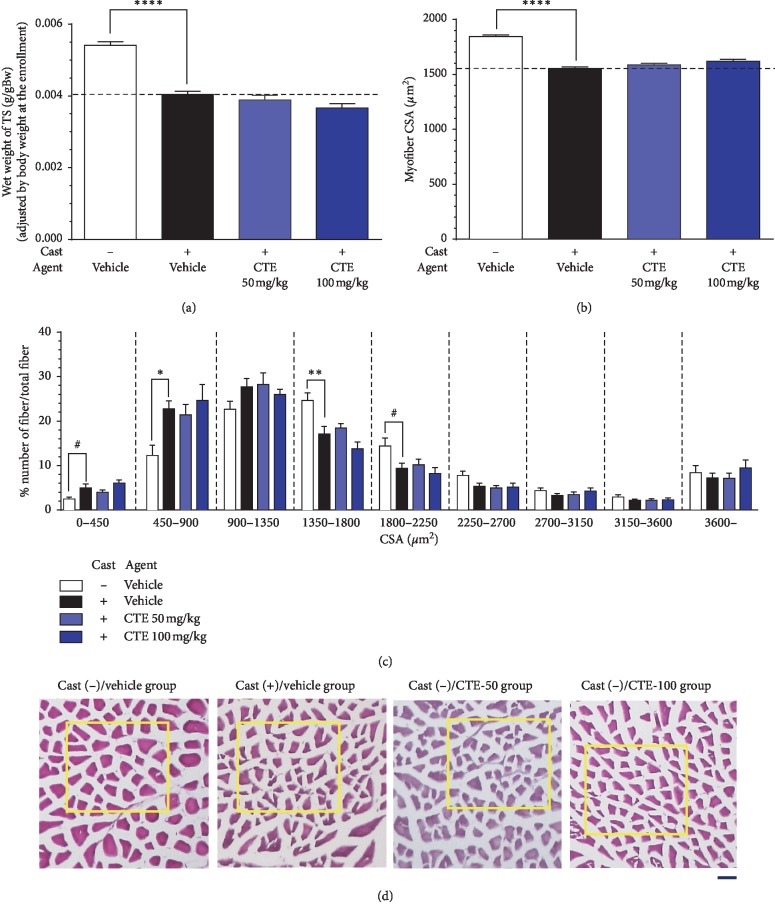
Morphometric group-wise characterization of murine triceps surae muscles (a)–(d). (a) Muscle wet weights standardized to body weight at the beginning of the experiment. (b, c) Effects of cast immobilization and CTE administration on myofiber cross-sectional area (CSA): mean CSA per group (b) and CSA distribution (c). (d) Representative schemas of TS myofibers via H-E staining in each group. The most representative area of each schema (from the point of view of comparing CSA) is surrounded by a yellow rectangular frame. Data are shown as mean ± SEM. Number of myofibers: *n* = 6550 in the cast (−)/vehicle group, *n* = 5857 in the cast (+)/vehicle group, *n* = 4699 in the cast (+)/CTE-50 group, and *n* = 5002 in the cast (+)/CTE-100 group. Number of mice: *n* = 9–11. Statistical analysis was performed by one-way ANOVA and post hoc Dunnett's test (a), Tukey's HSD test (b), or Kruskal–Wallis test with post hoc Dunn's test (c). ^*∗*^*p* < 0.05, ^*∗∗*^*p* < 0.01, ^*∗∗∗∗*^*p* < 0.0001, and ^#^*p* < 0.10 vs. cast (+)/vehicle group. Scale bar = 50 *μ*m.

The wet weight of the TS was significantly decreased with cast immobilization when compared with the cast (−)/vehicle group (Figure 4(a)). CTE administration did not influence TS wet weight.

The mean myofiber CSA in each group was also analyzed. In the cast (+)/vehicle group, a significant reduction in myofiber CSA was found compared with the cast (−)/vehicle group (Figure 4(b)). Myofiber CSA was further classified and analyzed by frequency distribution, as shown in Figure 4(c). The most frequent CSA class in the cast (−)/vehicle group was larger than that in the cast (+)/vehicle group; the percentage of the myofibers in the 1350‐2250 μm2 CSA class was, or tended to be, significantly higher in the cast (−)/vehicle group than in the cast (+)/vehicle group, and the opposite trend was apparent in the CSA class 0−900 *μ*m^2^ group (Figure 4(c)). CSA distribution was not influenced by CTE administration in the two cast (+)/CTE groups, as compared with the cast (+)/vehicle group (Figure 4(c)). Representative myofiber images are shown in Figure 4(d).

### 3.4. Effect of CTE on MyHC IId/IIx Expression Levels in the TS

**Figure 5:**
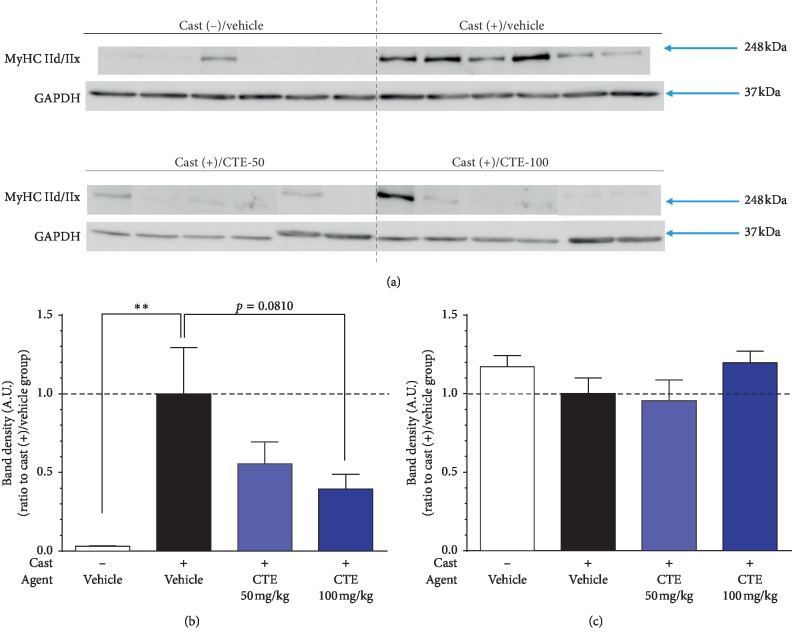
MyHC IId/IIx protein quantification in the TS muscle in each group (a)–(c). (a) Western blot of MyHC IId/IIx and GAPDH. (b) Densitometric measurements of MyHC IId/IIx at the end of the experiment. (c) Densitometric measurements of GAPDH (loading control) at the end of the experiment. The Smirnov–Grubb's test was used to exclude outliers and one-way ANOVA for analysis. Data are shown as mean ± SEM (*n* = 5–6). ^*∗∗*^*p* < 0.01 vs. cast (+)/vehicle group.

Western blotting was performed in six representative samples from each group (Figure 5(a)). These samples were selected based on their derivation from animals that exhibited foot-fault test performance closest to the mean values for their respective groups.

In the cast (+)/vehicle group, MyHC IId/IIx was significantly increased compared with the cast (−)/vehicle group. This increase was dose dependently suppressed by CTE administration. In the cast (+)/CTE-100 group, MyHC IId/IIx expression tended to decrease compared with the cast (+)/vehicle group (*p*=0.0810) (Figure 5(b)). GAPDH expression, which was used as an internal control, was not significantly different between groups (Figure 5(c)).

## 4. Discussion

Our novel cast immobilization method resulted in prominent muscle wasting, comparable to other cast immobilization methods [17–24], inducing approximately 10–45% of gastrocnemius muscle mass loss in 10 to 21 days. Also, myofiber CSA was decreased, indicating a reduction in muscle mass. This muscle change was accompanied by an increase of MyHC IId/IIx expression, which has been observed in several other studies of disuse muscle atrophy [11, 12]; hence, we believe that hindlimb disuse due to cast immobilization induced hindlimb muscle wasting.

The mammalian skeletal muscle contains slow-twitch and fast-twitch muscle fibers. The properties of these fibers are determined by the expression of multiple isoforms of MyHC (I*β*, IIa, IId/IIx, and IIb, which exists only in small mammals). Isoform I*β* has a slow-twitch profile, while isoforms IIa, IId/IIx, and IIb, in this order, have increasingly fast-twitch profiles, IIb being the quickest [36]. When muscle wasting occurs, a shift in the myofiber type also takes place, and, specifically, a slow-to-fast shift occurs in cases of loss of neural influence and mechanical loading. The changes in MyHC IId/IIx expression mentioned above are assumed to be a consequence of the myofiber type shift from slow to fast [10]. In contrast, malnutrition, inflammation, and sarcopenia induce a fast-to-slow myofiber type shift [10] through the reduction of type II fibers [1]; however, several reports revealed that type I fibers are also reduced in sarcopenia, where they form MyHC-coexpressing fibers [37].

Oral administration of CTE for 13 days tended to improve the deterioration of hindlimb function and to suppress the increase of MyHC IId/IIx expression, although not in a statistically significant way. Interestingly, muscle mass reduction did not simultaneously improve. These results suggest that CTE can attenuate the slow-to-fast myofiber type shift, thus contrasting the deterioration of hindlimb muscle function.

Since the TS presents naturally a slow-type contraction profile, it was suggested that slow-to-fast muscle fiber type shift along with muscle atrophy can induce impairment of muscle function, and an attenuation of this shift can mitigate it. For instance, Desaphy et al. reported that in the soleus muscle of hindlimb-unloaded mice, MyHC IId/IIx and IIb expression level increased, whereas that of IIa was decreased, and these changes occurred together with a shift of the mechanical threshold and alterations of the excitability parameters, along with muscle mass reduction; it was also revealed that antioxidant treatment by Trolox could neutralize these changes, but there was no effect on muscle mass [11]. Ferraro et al. also revealed a similar phenomenon using trimetazidine (TMZ); TMZ administration in aged mice improved forelimb muscle strength without increasing muscle mass, and this change was accompanied by an increase in MyHC I*β* [33]. In contrast, 8-week administration of *β*2-adrenoreceptor BRL-47672 significantly increased MyHC IIb, significantly decreased MyHC I*β* expression, and led to a 22% increase of gastrocnemius-plantaris-soleus muscle complex (GPS) mass, although it resulted in the deterioration of GPS performance [38].

The molecular mechanisms underlying CTE's effects on muscle wasting remain unknown, including the molecular components of CTE that affect muscle performance. We recently reported that at least one of the constituents of CTE, acteoside, ameliorates muscle performance of a mouse model of spinal-cord injury [39]. Several molecular pathways are presumed to be involved in the myofiber phenotype shift from slow to fast, including the Ca^2+^−calcineurin−nuclear factor's activation of the T-cell 1 (NFATc1) pathway and the Ca^2+^−calmodulin kinase-histone deacetylase (HDAC) 4/5−myocyte enhancement factor 2 pathway [36, 40, 41]. Additionally, pathways involving extracellular-related kinase (ERK) 1/2, AMP-activated protein kinase (AMPK)−sirtuin 1−peroxisome proliferator-activated receptor gamma coactivator 1-*α* (PGC1-*α*), and muscle-specific microRNAs are also related to myofiber type changes occurring with muscle disuse [36, 41]. However, no data are currently available about the relationship between CTE administration and myofiber type shift. Further experiments should seek to clarify the molecular mechanisms underlying the effects of CTE, as well as acteoside, on myofiber phenotype shifts and improvements in muscle performance; for this purpose, experiments on myoblast cell lines, such as C2C12 cells, should be performed [42].

Several other experiments should also be performed in future investigations of this subject. In order to robustly determine the effect of CTE on the myofiber type shift, CTE should be administered to various animal models of disuse muscle atrophy and tested, for example, on the diaphragm of mechanically ventilated animal [43] or on rats exposed to hypogravity by spaceflight [44]. Human data, such as in muscle atrophy due to bed rest, would also be necessary. Durations of CTE administration longer than 13 days should be tested, as they might lead to superior outcome. Moreover, CTE administration after inducing muscle atrophy may also have an effect on the recovery phase of muscle atrophy, while our data are focused only on its preventive effect.

## 5. Conclusions

Our results indicate that CTE attenuates the decrease in MyHC IId/IIx expression and improves the performance of the atrophied muscle caused by disuse, without increasing muscle mass. Furthermore, they demonstrate that the novel casting method for hindlimb immobilization introduced here is both effective and useful in muscle wasting experiments. We found that it induced a reduction in muscle size, muscle CSA, and MyHC IId/IIx content, along with impaired muscle performance.

## Data Availability

The data used to support the findings of this study are included within this article.

## Abbreviations

AMPK: AMP-activated protein kinase
CSA: Cross-sectional area
CTE: *Cistanche tubulosa* Schenk (Wight) extract
EPDM: Ethylene propylene diene monomer
ERK: Extracellular-related kinase
GPS: Gastrocnemius-plantaris-soleus muscle complex
GSK3*β*: Glycogen synthase kinase-3*β*
HDAC: Histone deacetylase
H-E: Hematoxylin-eosin
HRP: Horseradish peroxidase
MEF2: Myocyte enhancer factor 2
MyHC: Myosin heavy chain
NFATc1: Nuclear factor's activation of the T-cell 1
PGC1-*α*: Peroxisome proliferator-activated receptor gamma coactivator 1-*α*
PVC: Polyvinyl chloride
SDS-PAGE: Sodium dodecyl sulfate-polyacrylamide gel electrophoresis
Sirt1: Sirtuin 1
SEM: Standard error of the mean
TMZ: Trimetazidine
TS: Triceps surae muscle.
